# Editorial: The Brassicaceae — agri-horticultural and environmental perspectives, volume II

**DOI:** 10.3389/fpls.2022.1108246

**Published:** 2022-12-20

**Authors:** Naser A. Anjum, Sarvajeet Singh Gill, Om P. Dhankher, Narendra Tuteja, Juan F. Jimenez

**Affiliations:** ^1^ Department of Botany, Aligarh Muslim University, Aligarh, India; ^2^ Stress Physiology and Molecular Biology Lab, Centre for Biotechnology, Maharshi Dayanand (MD) University, Rohtak, India; ^3^ Stockbridge School of Agriculture, University of Massachusetts Amherst, Amherst, MA, United States; ^4^ Plant Molecular Biology Group, International Centre for Genetic Engineering and Biotechnology (ICGEB), New Delhi, India; ^5^ Institute for Scientific and Technological Research, San Luis Potosí, Mexico

**Keywords:** brassicaceae family, agricultural perspective, horticultural perspective, environmental health, plant breeding, crop improvement

Brassica is an old world genus and a monophyletic group within the Brassicaceae. Comprising about 35 species, the genus Brassica is represented by six interrelated cultivated brassicas, *Brassica napus*, *B. rapa*, *B. juncea*, *B. oleracea*, *B. nigra*, and *B. carinata*. Among the 18 research articles published in this volume II of the Research Topic ‘*The Brassicaceae - Agri-Horticultural and Environmental Perspectives*’, *B. napus*, *B. rapa*, *B. juncea*, and *B. oleracea* were researched in 11, 4, 2, and 1 articles on these respective genus.

This second volume provides insights into the molecular-genetic nature of the least and/or unexplored aspects in the major species of genus Brassica to date. It includes: (i) the quantitative trait loci related to silique-associated traits (Zhao et al.); (ii) heterosis of yield-influencing traits (Aakanksha et al.); (iii) basis of flowering time variations (Fang et al.); (iv) role of high-throughput phenotyping role in enlightening complex traits (Ebersbach et al.); (v) flower color and pigment formation (Li et al.; Hao et al.; Yang et al.); (vi) harvest index process and mechanisms (Zhang et al.); (vii) factors responsible for control of seed development stages and yield in special eco-environments (Xiong et al.); (viii) N rate-Auxin-Floral Meristem crosstalk (Hao et al.); (ix) cold resistance (Wu et al.); (x) male sterile lines role for hybrid seed production (Dong et al.); (xi) a comprehensive analysis on simple sequence repeats markers (Xu et al.); (xii) genome-wide characterization of ovate family protein gene family (Liu et al.); (xiii) relation of seed number per silique and ovule number per ovary with ovule fertilization and seeds development (Qadir et al.); (xiv) minimization of erucic acid in seed oil (Gill et al.); (xv) loci of clubroot-resistant genes (Wang et al.); and (xvi) functional characterization of the receptor-like proteins under the stimulus of *Sclerotinia sclerotiorum* (Li et al.).

Brief highlights and the major outcomes of the studies on *Brassica napus*, *B. rapa*, *B. juncea*, and *B. oleracea* included in this Research Topic are outlined below.

## Brassica napus

As the third-largest oilseed crop worldwide, rapeseed (*Brassica napus*) exhibits significantly high oil production efficiency. *B. napus* has evolved from the double diploidization of *B. rapa* and *B. oleracea* through interspecific hybridization. There has been a continuous search for factors contributing to improving *B. napus* yield. To this end, silique-related traits are of great significance. Analyzing the 120 consensus quantitative trait loci (QTLs) across multiple environments, 23, 25, 29, 22, and 21 consensus QTLs were identified for five silique-related traits (seed number per silique, SPS; silique length, SL; silique breadth, SB; silique thickness, ST; silique volume, SV), respectively (Zhao et al.). Interestingly, seed number per silique (SNPS) largely rely on the ovule number per ovary (ONPO), the proportion of ovules to be fertilized and the proportion of fertilized ovules to develop into seeds. ONPO widely varies in *B. napus*; however, underlying genes and mechanisms were unveiled and found to involve 18 novel association loci and six candidate genes (*BnaA03g14600D*, *BnaA03g33420D*, *BnaA06g08920D*, *BnaA06g13210D*, *BnaC01g25840D*, and *BnaC03g16210D*) (Qadir et al.). *B. napus* suffers from low genetic diversity; hence, exploitation of diverse genetic resources is imperative to develop locally adapted, high yielding, and stress resistant cultivars. To this end, it has been suggested that high-throughput phenotyping (HTP), an automated, scalable, non-destructive, and high-throughput imaging approach, is feasible for phenotyping studies on complex traits such as drought stress resistance, flowering characteristics (e.g., timing and volume), and canopy architecture traits (e.g., raceme branch numbers) in spring-type *B. napus* lines. These were selected as founders for the development of a large, spring-type *B. napus* Nested Association Mapping (NAM) population called “SKBnNAM” (Ebersbach et al.). The yield-loss in *B. napus* in some regions of the world also involves early frost-caused early flowering (maturation), which is often associated with flowering time (FT). Three genes (*BnFLC.A2*, *BnFLC.C2*, and *BnFLC.A3b*) were identified and argued to be major determinants for FT-variation of two elite *B. napus* accessions (616A and R11). Moreover, exploration of heterosis in FT-genes revealed a relation between FT and plant yield with hybrid near-isogenic lines (NILs) (Fang et al.).

In Brassica species, the flower color is among the most important traits contributing to pollen transmission in nature, and ornamental and landscaping purposes. Though cloned in tomato, maize, melon, and *Arabidopsis thaliana*, report on carotenoid isomerase (CRTISO) gene, responsible for converting the yellow-colored prolycopene into the red-colored all-trans lycopene in the carotenoid synthesis pathway is lacking in *B. naups*. Notably, as the most simple and efficient sequence-specific nucleases (SSNs), CRISPR/Cas9 (Li et al.) helped edit two copies of the carotenoid isomerase gene (*BnaCRTISO*) in *B. napus* (*BnaA09.CRTISO* and *BnaC08.CRTISO*), recovering the mutation phenotype of creamy white petals and yellowish leaves, and establishing a correlation between the carotenoid pathway and flavonoid synthesis pathway, so far not well known in most plants. Information is meager on the major mechanism underlying the involvement of the anthocyanin pathway (crucial in plant color development, ranging from pink to blue and purple) in *B. napus* petal color formation. An investigation employing metabolomics and RNA-seq studies on two different stages of unopened petals of red, pure white, and yellow petal *B. napus* lines identified *BnaA03.ANS* as the gene involved in *B. napus* petal color control (Hao et al.).

Harvest index (HI) is a complex agronomic trait and an economically critical value, representing the ratio of harvested seed weight to total aboveground biomass weight. Unfortunately, *B. napus* exhibit values of HI that are much lower than in other major crops, and the molecular-genetic regulatory network underlying HI in *B. npus* is largely unknown. Interestingly, studies on the *B. napus* accessions YC24, YC52, and YC46, exhibiting differential HI, mRNA, and small RNA sequencing revealed the role of transporter activity-related genes in enhancing HI under good cultivation environments (Zhang et al.). The development of seeds (which comprises three major stages: sugar mobilization, sequential surges in amino acid, lipid, and storage protein synthesis) is largely modulated by environmental factors in a special eco-environment exhibiting differential sunshine duration, temperature, and water. Notably, when cultivated under such a special eco-environment, *B. napus* (Qingza 5, a spring rapeseed variety)-grain development was found to be ahead of schedule, which lasted for a long time, and led to a high-yield (Xiong et al.). The production of *B. napus* is significantly modulated by conversion from vegetative to reproductive growth, which in turn is determined by the acceleration of the differentiation of floral meristem (FM) from shoot apical meristems (SAM). SAM differentiation can be regulated by many factors including phytohormones (such as auxin), and environmental and agronomic practices such as low temperature and nutrients (such as N). Interestingly, a high N rate can accelerate the initiation of FM differentiation, and involve auxin, which in turn involves both auxin biosynthesis genes (*ASA1/ASA2*, *IGS*, *TSA1*, *TSB1*, *CYP79B*, *NIT1*/*NIT2*, and *AMI1*) and also indole acetaldehyde oxime pathway as the major pathway for auxin biosynthesis (Hao et al.). *B. napus* has experienced whole genome duplication and shares significant homology with *Arabidopsis thaliana*, known to possess multiple copies of genes of ovate family proteins (OFPs; AtOFP1, AtOFP4, AtOFP5). However, B. napus was not been reported to exhibit any function of OFPs. The genome-wide characterization of the OFP gene family associated with the number of seeds per silique (NSPS) in B. napus helped in the identification of both the BnOFP gene family at the genomic level and a new locus *BnOFP13_2*, which was significantly correlated with NSPS (Liu et al.). Among the major multiple external stimuli, *Sclerotinia sclerotiorum* has emerged as an important Brassica pathogenic fungi also known to remarkably affect the yield and quality of *B. napus*. A comparison of the transcriptional profiles of genes of receptor-like proteins (RLPs, an indispensable constituent in the first layer of defense, and typically with tandem leucine-rich repeats) with that of the functional assigned *AtRLP* genes revealed *AtRLP10* (CLV2, AT1G65380.1) as a regulator of plant meristem and organ development, and *S. sclerotiorum*-exerted stimulus-caused decrease in transcript abundance of *BnaC02g45200D* and *BnaA02g12070D* (Li et al.).

## Brassica rapa

One of the three diploid ancestors of *B. napus* and *B. juncea*, *B. rapa* is highly diverse, and of nutritional and economic importance as it is widely cultivated worldwide as an oil and vegetable crop species. Few studies have reported on the genes underlying the dark yellow flower trait in Chinese cabbage (*B. rapa* L. ssp. *Pekinensis*). To this end, *Bra037130* (*BraA09.ZEP*) (which encodes a zeaxanthin epoxidase) has been argued to be the most likely candidate gene for *Br-dyp1* involved in the epoxidation from zeaxanthin to violaxanthin in *B. rapa* L. ssp. Pekinensis (Yang et al.). The health and yield of *B. rapa* (winter rapeseed) are lagging due to chronic low temperatures in winter in some regions of the world such as northwest China. Elucidation of candidate genes associated with cold resistance in *B. rapa* has been limited by currently existing genomes. Taking into account a freeze-tolerant winter turnip variety “Longyou-7” (LY7), assembly of its high-quality genome (using PacBio HIFI reads) and construction of a graph-based pan-genome by combining LY7-genome with the other 22 *B. rapa* accessions led to the identification of *HDG1* and *BrANS3* as the two genes associated with cold tolerance (Wu et al.). Breeding of male sterile lines is crucial for the hybrid seed production and commercialization of *B. rapa* L. ssp. *Pekinensis*, which is a typical cross-pollinated Brassica crop with obvious heterosis. Interestingly, three allele male sterile mutants (*msm2-1/2/3*) were identified in an ethyl methane sulfonate (EMS)-mutagenized *B. rapa* L. ssp. *Pekinensis*, where pollen abortion was obtained due to non-synonymous base-pair mutations in BrMS1 (*BraA10g019050.3C*), and *AtMS1* orthologs gene (relevant to fertility and pollen development regulation) was cloned first time in EMS-mutagenized *B. rapa* L. ssp. *Pekinensis* (Dong et al.).

The life of most Brassica species (including *B. napus*, *B. oleracea*, and *B. rapa*) is endangered by a highly contagious soil-borne disease, clubroot (caused by *Plasmodiophora brassicae*). Exploration is imperative to identifying new resources for resistance genes owing to reports on the loss of resistance over time and the presence of various pathotypes for clubroot-resistant (CR) pathogens from different regions. Notably, DingWen (DW) identified a unique *B. rapa* material that shows different resistance capabilities from H5R (PbBa8.1) and Huayouza62R (CRb) in *B. napus*. Moreover, the peculiar resistance in DW most likely involves the CR candidate genes *BraA08g039211E* and *BraA08g039212E* (encoding TIR-NBS-LRR proteins), and *BraA08g039193E* (encoding an RLP protein), which could also act as a new gene resource for CR breeding in the future (Wang et al.).

## Brasica juncea

Indian mustard (*B. juncea*) is a major oilseed crop in the south Asia region, including the Indian sub-continent. Unfortunately, *B. juncea* seed oil exhibits a very high concentration (≈50%) of erucic acid, which, if continuously consumed, may cause cardiac lipidosis. KASPar assay, when developed to select low erucic acid and to track recessive alleles in their heterozygous state, is single step, has a high throughput, and is robust, codominant, and more cost-effective than gel-based markers (Gill et al.). The heterotic quantitative trait loci (QTL) has been mapped and a large number of epistatic interactions were identified for seed yield and three yield-component traits in *B. juncea* using doubled haploids (DH) and the corresponding backcross lines with their midparent heterosis data. However, the molecular mechanism underlying the complex phenomenon, particularly in *B. juncea* is not well understood. Fortunately, the complex genetic basis of heterosis of yield-influencing traits (plant architecture, flowering, and silique- and seed-related traits) was unveiled in *B. juncea* and argued to involve a large number of additive QTLs (695) and loci (637 epistatic loci). It also exhibited cumulative effects of dominance, overdominance, and a large number of epistatic interactions (Aakanksha et al.).

## Brassica oleracea

As one of the most critical cruciferous vegetables, cabbage (*B. oleracea* L. var. *capitata*) has been widely cultivated all over the world since it contains favorable components for human health. Notably, compared with the development of simple sequence repeats (SSR) markers in other crops, SSR markers, those developed from *B. oleracea* L. var. *capitata* reference genome are lacking. Additionally, despite the contribution of RNA-Seq data to in silico generation, a comprehensive analysis of SSRs within *B. oleracea* L. var. *capitata* genome is not available. To fill the highlighted knowledge-gaps, 64,546 perfect and 93,724 imperfect SSR motifs were identified in the 0.5Gb of *B. oleracea* L. var. *capitata* genomic sequence (TO1000), which was mined using a whole-genome bioinformatics survey (Xu et al.).

## Conclusions and outlook

The contributions to volume II of the Research Topic ‘*The Brassicaceae - Agri-Horticultural and Environmental Perspectives*’ discussed the molecular-genetic insights into agri-horticultural aspects that can help in devising breeding approaches in various species of the genus Brassica for yield, flowering time variations, flower color, and pigment formation. The collection discusses the factors involved in seed development stages and yield in special eco-environments, N rate-Auxin-Floral Meristem crosstalk, resistance to cold and pathogens, hybrid seed production, and minimization of erucic acid in seed oil. However, no contributions discuss the molecular-genetic aspects of abiotic stress tolerance and the environmental perspectives of the Brassicaceae family members. Additionally, there is a large number of existing research on the contribution of the members of the plant family Brassicaceae in the control of varied environmental issues. These highlighted aspects will be further explored in future volume(s) of this Research Topic.

## Author contributions

NA prepared the first draft of the manuscript. SG OD, JJ, and NT read and revised the manuscript, and all authors approved the final version for publication.

